# Divided Sanitary Responsibility

**Published:** 1902-04-12

**Authors:** 


### The Hospital

A JOURNAL OF

The Medical Sciences and Hospital Administration.

VOL. XXXII.—NO. 811.] EDITED BY SIR HENRY BURDETT, K.C.B., AND SOLOMON C. SMITH, M.D., M.R.C.P. [APRIL 12, 1902.

### DIVIDED SANITARY RESPONSIBILITY.

A DEPUTATION which was received by the President of the Local Government Board last month gave an account of the very serious extent to which the small-pox hospitals belonging to the Metropolitan Asylums Board have acted as centres for the diffusion of the very disease for the control of which they were established. The facts are sufficiently clear and the teaching is sufficiently obvious. Sir William Hart Dyke said that there had been in the Dartford Urban District 190 cases of small-pox, of which no fewer than 117 were directly traceable to the hospitals of the Asylums Board. The rural district of Dartford had 60 cases, many of them traceable to the workpeople engaged at the hospitals; Bexley Urban District had 45 cases, Erith 195, Northfleet 34, Gravesend 35, and a considerable number of all these cases were in each instance traceable to the hospitals. It was evident that a large number of workpeople had been engaged upon the erection of the temporary hospitals, in other words, had been employed in the neighbourhood of wards occupied by small-pox patients without any effectual steps having been taken to secure their immunity from small-pox by means of vaccination. So much for the bare facts of the case. They are bad enough, and the laxity they display would seem almost without excuse even if nothing more were at the back of it. We cannot but feel, however, that there is a good deal at the back of it, and that the extraordinary indifference to the public welfare displayed in this unfortunate business is but one example of the many evils which are inherent in the bad system which has been permitted to take deep root in the sanitary organisation of this blundering metropolis.

In every other city the hospitals for the isolation of patients suffering from infectious diseases are under the direct management of those responsible for the public health—they are, in fact, part and parcel of the machinery devised and maintained with the object of controlling epidemics. London in its wisdom, however, has arranged that in its case this shall not be so. The metropolitan hospitals for infectious diseases are managed by a body which has no concern and no responsibility in regard to the control of epidemics. If this body deals well with the patients whom it receives its duty is done and no more can be demanded of it. If it chooses to fill up its wards with patients from an area in which an epidemic has taken such root that it is hopeless to attempt to control it, and if then, its wards being full, it refuses a case which, being the first patient attacked in a new district, ought from a public health point of view to be isolated at any cost, there is no one to say no. It can fold its hands and maintain with perfect justice that when its wards are full and its patients are well treated its duty to the metropolis is performed, and that for all it cares London may simmer in its own juice. We do not say that in practice things are quite so bad as this, because we believe that, by the zeal of our medical officers and by their personal willingness to go beyond the strict limits of their duties, we are to some extent relieved of the consequences of our absurd system of sanitary organisation. The fact remains, however, that, by the multiplication of authorities and by the delegation of public health duties to independent bodies, the sanitary administration of the metropolis is hampered at every turn. At the present time London employs a small army of expert sanitary officials, and we will undertake to say that if they were canvassed as to what are the three principal means by which an outbreak of small-pox can be best controlled (putting on one side, of course, those ordinary sanitary measures which are always in operation) they would answer, almost without exception, first vaccination, second isolation, and third tracing out the sources of infection and quarantining "contacts." Yet, if asked further what is being done by them in the matter, they would have to confess that in regard to the first, they have done nothing, for that is a measure which is entirely taken out of their hands; that as to the second, although the responsibility still lies upon them by law, they in practice do nothing, the whole business being delegated to an independent body in which they have no voice or representation, and over whose proceedings they have no control; while, as to the third, they would have with sorrow to admit that the thing is impossible owing to London being cut up into such a multitude of independent sanitary areas. They may, and no doubt they do, report to neighbouring sanitary authorities such facts as they discover, and these neighbouring authorities, no doubt, make just as much or as little use as they like of the knowledge so obtained. But there is no central body by which the infection can be tracked to its source, and by which the movements of "contacts" can be controlled.

It is almost inconceivable that such an absolute lack of unity should exist in the sanitary administration of our great metropolis; but such is the fact, and nothing could better illustrate the evil effects of such divided responsibility, and the extent to which the delegation of small sections of sanitary work to independent bodies may lead to the main objects of such work being overlooked, than the spectacle of this great city blindly paying hundreds of thousands of pounds to the Metropolitan Asylums Board in the hope of obtaining protection from small-pox, while this Board is all the while so spending this money as to ensure the wide diffusion of small-pox infection on every side. Yet the Board is within its rights. "What have we to do with Dartford?" it may say. "Our duty is towards our patients; let the Dartford people take care of themselves." This may be business, but it is the exact opposite of what would be done if the provision of isolation hospitals were regarded, as it should be regarded, as merely part of a great scheme for the control of infectious diseases.

